# Radiology medico-legal cases

**Published:** 2008-05

## From *Medical Law Cases - For Doctors,* Vol 1: 1; pgs 34, 35

## Case 1: Medical Literature in Aid to Justify Medical Action

Editor's note: The article has been modified from the original to protect the identities of those concerned.

## Summary

Dr. A and Ors versus Patient Z; Judgment in favor of: Doctor.

### Facts of the case

The patient underwent a USG examination of the abdomen, performed by the sonologist (Opposite Party - OP) and the report read, “gall bladder …. contains multiple echogenic shadows of stones accompanied by P-A shadowing in the neck region.” Relying on the report, the patient was operated for removal of stones but no stones were found.

### Patient's allegation/s of medical negligence

The allegation was that a wrong report was given by the sonologist (OP) and relying on the same, the patient underwent surgery. It was alleged that it was unconvincing that stones could have disappeared within a period of 18 days of the USG, i.e., when the surgery done.

## Doctor's Defense

- The doctor in his defense produced medical literature wherein it was categorically stated, “Finally small gallstones are occasionally seen to disappear…. This can happen at any time and is not necessarily accompanied by symptoms.”
- Further, the operational notes of surgeon recorded, “Gall bladder had few adhesions with omentum and the cut surface showed thickened mucosa with few tiny whitish spots? Calcareous deposits, but did not contain any calculi” were also pointed out in defense.

## Findings of the Court

- The court in view of the medical literature, the operating surgeon's statement in court and findings recorded in the operation notes, held that it was possible for stones to disappear within 18 days.
- Hence, the sonologist (OP) was not found to be negligent.

## Detailed Discussion

1. The appellants were the opposite parties before the State Commission, where the respondent/complainant, patient Z had filed a complaint alleging a case of medical negligence against the appellants.
2. Very briefly the facts of the case are that the respondent/complainant got a USG examination of the abdomen done by the appellants for a consideration and report was supplied to the complainant. The report reads as follows:
  “The gall bladder is normal in size and shape. Its wall thickness is 3 mm. It contains multiple echogenic shadows of stones accompanied by P-A shadowing in the neck region. No mass seen. No perifocal edema seen. No stone seen in the CBD or cystic duct.”
3. Because this report showed multiple shadows of stones, the complainant got herself admitted for removal of stones and operation was carried on, on 11.6.1996 by one Dr. B, but in reality, no stones were found. Thus, alleging a case of medical negligence, a case was filed before the State Commission, who after hearing the parties allowed the complaint and directed the appellants to refund Rs. 400 charged for the examination along with cost of Rs. 1,000. Aggrieved by this order, this appeal has been filed before us.
4. Despite service of notice, none appears for the respondent/complainant, hence proceeded *ex parte*. We heard the appellant Dr. C on behalf of the appellants.
5. After hearing appellant and perusal of material on record as well as the medical literature brought on record, we find that the State Commission has held the appellants guilty of medical negligence on account of the fact that while the USG test carried out on 24.5.1996 by the appellants showed multiple echogenic shadows of stones in gall bladder by P-A shadowing in the neck region, the State Commission found it unconvincing that it could have disappeared within a period of 18 days, i.e., when the surgery done by Dr. B on 11.6.1996, the stones were not seen.
6. The appellant has brought our attention to the medical literature titled “*A Textbook of Radiology and Imaging*” edited by David Sutton 3rd edition, in which it is laid down as follows:
  “Finally small gallstones are occasionally seen to disappear. This is usually due to their passage into b the common duct where they mayor may not be retained. This can happen at any time [Figure 37.6] and is not necessarily accompanied by symptoms. This is one of the main justifications for operative choledochography.”
7. Our attention has also been drawn towards the operational notes of Dr. B, on which the findings have been recorded in following terms:
  “Gall bladder had few adhesions with omentum and the cut surface showed thickened mucosa with few tiny whitish spots? Calcareous deposits, but did not contain any calculi. Liver and other adjacent viscera normal. CBD normal. Gall bladder sent for HPE (Histopathological examination).”
8. We have also seen the cross-examination of the concerned surgeon, wherein he has clearly admitted, “…if the stone is of very small size or few in number, there could be its passing out during the period between 24.5.96 and 11.6.1996.”
9. In view of the medical literature brought on record cited above and what the operating surgeon stated in his cross-examination, along with his findings referred to in the operation notes, we are of the view that in the light of this material, the order passed by the State Commission cannot be sustained. There is neither any other expert evidence nor material brought on record by the respondent/complainant in support of the allegations of medical negligence on the part of the appellants. Only, deemed to be expert, Dr. B has admitted that these stones could have passed out during this period. In view of this, according to us, the State Commission could not have held the statement of the appellants “not at all convincing”.
10. In the aforementioned circumstances, we are unable to sustain the order passed by the State Commission, which is set aside. The appeal is allowed and the complaint stands dismissed.
11. No order as to costs.
12. Appeal allowed.

